# Sentinel Lymph Node Mapping Compared with Selective Lymph Node Sampling in the Surgical Staging of Endometrial Cancer: A Prospective Observational Study

**DOI:** 10.3390/diagnostics16121904

**Published:** 2026-06-19

**Authors:** Vlad Alexandru Gâta, Radu Alexandru Ilieș, Ana Maria Mureșan-Bădescu, Ștefan Țîțu, Alexandra Timea Kirsch-Mangu, Anda Gâta, Delia Nicoară, Alexandra Sîncrăianu, Boutaina Chakir, Florin Laurențiu Ignat, Ioan Cătălin Vlad, Alexandru Irimie, Patriciu Andrei Achimaș-Cadariu

**Affiliations:** 1 Department of Oncological Surgery, “Iuliu Hațieganu” University of Medicine and Pharmacy, 400012 Cluj-Napoca, Romania; vlad.gata@umfcluj.ro (V.A.G.); fignat2003@yahoo.com (F.L.I.); catalinvlad@elearn.umfcluj.ro (I.C.V.); pachimas@umfcluj.ro (P.A.A.-C.); 2 Department of Oncological Surgery and Gynecological Oncology, “Prof. Dr. Ion Chiricuță” Institute of Oncology, 400015 Cluj-Napoca, Romania; stefan.titu@umfcluj.ro (Ș.Ț.); alexandralupsa25@gmail.com (A.S.); boutaina.cr@gmail.com (B.C.); airimie@umfcluj.ro (A.I.); 3 Faculty of Medicine, “Iuliu Hațieganu” University of Medicine and Pharmacy, 400012 Cluj-Napoca, Romania; ilies.radu.alexandru@elearn.umfcluj.ro (R.A.I.); 4 Department of Oncology, “Iuliu Hațieganu” University of Medicine and Pharmacy, 400012 Cluj-Napoca, Romania; timeakirsch@gmail.com (A.T.K.-M.); 5 Department of Radiation Oncology, “Prof. Dr. Ion Chiricuță” Institute of Oncology, 400015 Cluj-Napoca, Romania; 6 Department of Otorhinolaryngology, “Iuliu Hațieganu” University of Medicine and Pharmacy, 400012 Cluj-Napoca, Romania; 7 Department of Quality Management, “Prof. Dr. Ion Chiricuță” Institute of Oncology, 400015 Cluj-Napoca, Romania; drdelianicoara@gmail.com (D.N.)

**Keywords:** endometrial cancer, sentinel lymph node, lymph node assessment, indocyanine green, methylene blue, lymph node metastasis, surgical staging, gynecologic oncology

## Abstract

**Background/Objectives**: Nowadays, lymph node assessment represents a key component in the surgical staging of endometrial cancer, as sentinel lymph node (SLN) mapping increased in adoption as an alternative to lymphadenectomy. This study aimed to compare SLN mapping and selective lymph node sampling (SLNS) in endometrial cancer cases managed in a tertiary oncologic center. Moreover, the study evaluated clinicopathological characteristics and the association between tumor stage and nodal involvement. **Methods**: This prospective observational cohort study included 137 patients with histologically confirmed endometrial cancer who underwent surgical staging between January 2020 and August 2025. Either SLN mapping using indocyanine green (ICG) or methylene blue (blue dye–BD) or SLNS was performed during the surgery. Clinical, surgical, and histopathological data were analyzed using descriptive and inferential statistics. **Results**: SLN mapping was performed in 86 patients (BD: 45; ICG: 41), while the other 51 underwent SLNS. Median lymph node yield was significantly higher in the SLNS group (10 nodes) in comparison to SLN mapping (four nodes for both BD and ICG; *p* < 0.001). The overall nodal metastasis rate was 9.5%, with no significant difference between techniques (SLNS: 9.8%, BD: 8.9%, ICG: 9.8%; *p* = 0.99). Bilateral nodal detection rates were higher in the BD group compared to the ICG group (73.3% vs. 51.2%; OR = 2.62, *p* = 0.045). Nodal involvement increased significantly in parallel with advancing pathological T stage (*p* < 0.001), ranging from 0% in T1a to 40.0% in T3a disease. **Conclusions**: Even though SLNS resulted in a higher number of lymph nodes retrieved, SLN mapping demonstrated similar observed rates of nodal metastases across groups within the limits of this observational study. BD demonstrates superior bilateral detection rates compared to ICG in this cohort. Tumor stage remains a predictor of lymph node involvement. All these findings justify the use of SLN mapping as an effective staging strategy in patients with endometrial cancer.

## 1. Introduction

Recent epidemiologic data demonstrate a consistent global increase in endometrial cancer incidence. The age-standardized incidence rate increased by 0.69% per year regardless of sociodemographic status. In the United States, type 1 endometrial cancer incidence increased dramatically across all age groups, with 14-fold, 63-fold and 50-fold increases among women aged <45, 45–54, and ≥55 years. The mean age at diagnosis decreased from 64.1 to 61.0 years [1,2]. However, while global age-standardized mortality rates decreased by 0.85% annually, the mortality-to-incidence ratio remains higher in developing countries [1,2].

Obesity emerges as a key modifiable risk factor, with each 5 kg/m^2^ increase in BMI associated with a relative risk of 1.59 for developing endometrial cancer, and the rising obesity prevalence among younger women correlates temporally with increased incidence in this population [2]. These findings indicate that endometrial cancer represents a growing global health burden requiring enhanced prevention efforts.

Endometrial cancer is primarily managed with surgery. The cornerstone of treatment for early stage (stage I–II) endometrial carcinoma is total hysterectomy with bilateral salpingo-oophorectomy, preferably performed using minimally invasive techniques. For advanced disease (stage III and IV), surgical cytoreduction and suspicious lymph node resection should be considered when complete macroscopic resection is possible [3].

Sentinel lymph node biopsy (SLNB) is the recommended method for nodal staging in presumed uterus-confined disease (low-risk and intermediate-risk patients). Systematic lymphadenectomy is not recommended for low-risk disease. Side-specific lymphadenectomy is reserved for non-identifiable SLN on one side or for high-intermediate or high-risk patients [3].

Omentectomy is required for serous, carcinosarcoma and undifferentiated histologies, reflecting their aggressive biological behavior [3].

Ovarian preservation may be considered only in carefully selected young, premenopausal patients (<45) with low-risk FIGO stage IA endometrioid carcinoma and no hereditary risk factors [3].

For apparent early-stage endometrial cancer, total hysterectomy with bilateral salpingo-oophorectomy (TH/BSO) remains the backbone of surgical management. The controversy centers on how aggressively to assess lymph nodes [3,4,5].

Systematic pelvic lymphadenectomy improves staging accuracy, but does not improve disease-free or overall survival in unselected early-stage disease, while clearly increasing operative time and postoperative complications. There is a possible survival benefit of extensive lymphadenectomy only in selected high-risk or high-grade subsets and when para-aortic nodes are also addressed, but these data are subject to confounding [4,5].

However, lymphadenectomy is a major driver of morbidity. The extent of nodal dissection is linked to higher rates of lymphocele and lymphedema, longer operative times and increased blood loss, which can severely impact long-term quality of life. This has led to a shift in philosophy: lymph node surgery should provide reliable prognostic and treatment-guiding information while minimizing harm. In this context, complete pelvic (and para-aortic) lymphadenectomy is now generally reserved for patients with high-risk features (deep myometrial invasion, non-endometrioid histology, high grade, suspicious nodes or radiologic evidence of nodal involvement) or for settings where sentinel lymph node (SLN) mapping is not available or reliable [4,5].

SLNB is based on the concept that lymphatic spread from the uterus follows an orderly pattern: the first draining lymph nodes (“sentinel” nodes) reflect the status of the entire nodal basin. In practice, a tracer (dye, radiocolloid or fluorescent agent) is injected into the cervix or uterine fundus and SLNs are identified intraoperatively and examined with ultrastaging (serial sectioning and immunohistochemistry), allowing detection of micrometastases and isolated tumor cells [6,7,8].

SLN mapping in endometrial cancer has high detection and bilateral mapping rates and very low false-negative rates when the SLN algorithm is respected (removal of all mapped nodes, side-specific lymphadenectomy if mapping fails and ultrastaging) [6,7,8].

SLNB is considered appropriate for apparent stage I–II endometrial cancer, especially low- and intermediate-risk disease, and reasonable even in selected high-risk histologies (grade 3 endometrioid, serous, clear cell, carcinosarcoma) when a validated SLN algorithm and ultrastaging are applied, without compromising survival [6].

As there is no significant difference in recurrence or overall survival between SLN algorithms and comprehensive lymphadenectomy, SLNB is supported as an oncologically sound alternative for nodal staging. As a result, SLN mapping has transitioned from an experimental technique to a widely accepted standard of care for nodal assessment in early endometrial cancer in many centers [7,8].

Several tracer strategies have been used for SLN mapping in endometrial cancer. Blue dye (BD) (e.g., isosulfan, methylene blue) is a simple and inexpensive method, but limited by shallow tissue penetration, lower bilateral detection rates and rare but serious anaphylactic reactions. Technetium-99m radiocolloid (Tc-99m) allows preoperative lymphoscintigraphy and SPECT/CT to visualize drainage patterns, but requires nuclear medicine infrastructure, exposes staff/patients to radioisotopes and adds logistical complexity. Combined blue dye and Tc-99m has historically improved detection compared with either alone, but keeps the drawbacks of both. Indocyanine green (ICG) with near-infrared (NIR) imaging is now the dominant approach because it is well tolerated and serious reactions are rare, especially compared with BD–related anaphylaxis reported in the literature [9,10,11,12].

ICG consistently achieves higher overall and bilateral SLN detection compared to BD or Tc-99m protocols, with similar or better diagnostic accuracy. NIR-ICG mapping is superior or at least non-inferior to conventional tracers and is especially advantageous in minimally invasive surgery where fluorescence is easily visualized [9].

The current ESMO/ESGO guidelines recommend a nuanced approach to surgical management of endometrial cancer, with SLNB increasingly replacing comprehensive lymphadenectomy [13].

Oncologic safety appears comparable between the two methods. There are no significant differences in disease-free or overall survival between patients staged using an SLN algorithm and those undergoing comprehensive pelvic with or without para-aortic lymphadenectomy, including women with deeply invasive disease or high-risk histologic subtypes. Staging quality may be enhanced with SLNB, as the use of ultrastaging allows for more frequent detection of low-volume nodal metastases, improving risk stratification without adding surgical morbidity. Finally, morbidity is substantially lower, and adverse outcomes are markedly reduced with SLNB.

Ongoing and recent randomized or non-inferiority trials (e.g., ALICE, KGOG2029) further suggest that SLNB can safely replace full pelvic lymphadenectomy as the default nodal assessment in appropriately selected stage I–II endometrial cancer, with lymphadenectomy reserved for failed or unilateral mapping (as part of the SLN algorithm), grossly suspicious nodes or specific high-risk scenarios where comprehensive nodal clearance is still considered necessary by guidelines or trial protocols [14,15].

The main objective of the present study is to assess and compare lymph node assessment strategies in surgically staged endometrial cancer, with a particular focus on SLN mapping versus selective lymph node sampling (SLNS). Secondary objectives included description of the demographic, surgical and histopathological characteristics of the cohort and investigating the association between the pathological tumor stage and lymph node involvement. The study aims to clarify the staging performance of different lymph node evaluation approaches in a tertiary oncologic center setting.

## 2. Materials and Methods

### 2.1. Study Design and Patient Selection

This prospective observational study was conducted at the Institute of Oncology “Prof. Dr. I. Chiricuță”, Cluj-Napoca, Romania (IOCN), a tertiary center for gynecologic oncology. The study included consecutive patients who were diagnosed with endometrial carcinoma and underwent surgical staging between January 2020 and August 2025.

**Figure 1:**
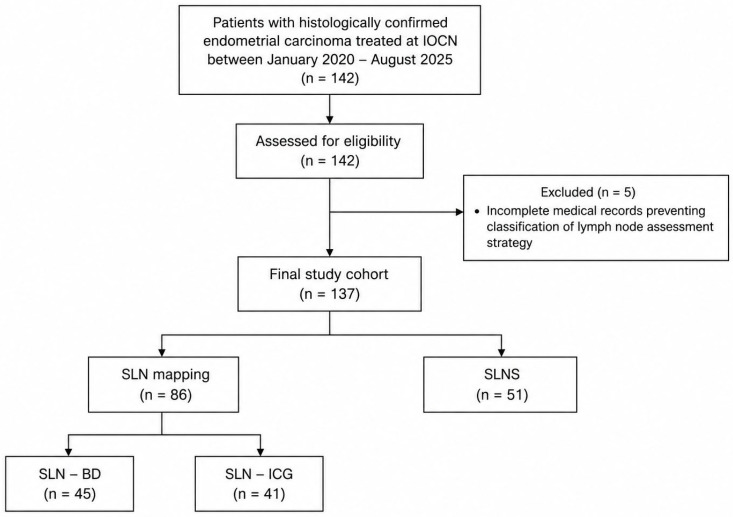
Flowchart illustrating the study design, patient selection and lymph node assessment strategy. IOCN = Institute of Oncology “Prof. Dr. I. Chiricuță”, Cluj-Napoca, Romania; SLN = sentinel lymph node; BD = blue dye; ICG = indocyanine green; SLNS = selective lymph node sampling.

Patients with endometrial cancer (that was histologically confirmed) who underwent surgical staging were found eligible. Patients with incomplete medical records that did not allow classification of lymph node assessment strategy were excluded. A total of 142 patients were initially identified for eligibility; 5 patients were excluded due to incomplete data necessary for lymph node assessment categorization, resulting in a final study cohort of 137 patients. The study design is summarized in Figure 1.

### 2.2. Lymph Node Assessment Strategy, Surgical Procedure and Pathological Evaluation

SLN mapping was performed using ICG as the primary tracer or BD as an alternative dye. The choice of tracer was based on institutional availability at the time of surgery. Comparative analyses between the two mapping techniques were subsequently performed.

For ICG mapping, a 25 mg vial of indocyanine green was reconstituted with 10 mL of sterile water for injection, yielding a final concentration of 2.5 mg/mL. A total volume of 4 mL (10 mg ICG) was administered per patient. Following induction of general anesthesia and prior to the start of the surgical procedure, cervical injection was performed at the 3 and 9 o’clock positions. At each site, the tracer was injected in two planes: a superficial subepithelial injection (approximately 1–3 mm depth) and a deep stromal injection (approximately 1 cm depth). Specifically, 1 mL was injected superficially and 1 mL deeply at each position, resulting in a total injected volume of 4 mL. Aspiration was performed prior to injection, and the dye was administered slowly to minimize intravascular injection and cervical reflux.

As an alternative technique, methylene blue was injected following the same cervical protocol (3 and 9 o’clock positions, superficial and deep planes, total volume of 4 mL). In these cases, SLNs were identified by direct visual inspection of blue-stained lymphatic channels and nodes, without fluorescence imaging.

The choice of tracer (blue dye versus indocyanine green) relied on institutional availability at the time of surgery. Both techniques were carried out during the same overall study period and were performed by surgeons experienced in minimally invasive gynecologic oncology using standardized surgical platforms. No predefined temporal separation was applied between the two tracer groups. This introduces a potential temporal and selection bias, as tracer availability may have varied during the study period.

Both open and laparoscopic surgical approaches were included. In open procedures, near-infrared fluorescence imaging was performed using the Artemis Surgical detection system, whereas in laparoscopic procedures fluorescence visualization was achieved using an integrated near-infrared imaging platform provided by Olympus (Tokyo, Japan). After abdominal access, lymphatic drainage pathways were assessed bilaterally, and all fluorescent or blue-stained SLN were excised and labeled according to laterality and anatomical station (e.g., obturator, external iliac, internal iliac).

The majority of patients included in the study had no preoperative clinical or radiological suspicion of lymph node involvement. Preoperative assessment included biopsy histology, tumor grade, imaging findings, estimated myometrial invasion depth, suspected cervical involvement, and overall clinical staging, which were integrated with intraoperative judgment when selecting the nodal assessment strategy.

SLN mapping was preferentially performed in clinically early-stage, uterus-confined disease.

In a subset of patients, selective lymph node sampling (SLNS) was performed instead of SLN mapping. This approach was chosen upfront at the discretion of the operating surgeon and was planned preoperatively, rather than being added intraoperatively to SLN mapping. SLNS was reserved for cases in which SLN mapping was not available or feasible, or when intraoperative findings prompted targeted nodal evaluation relying on surgeon judgment rather than systematic lymphadenectomy. This procedure did not follow a systematic pelvic lymphadenectomy template and consisted of selective removal of clinically suspicious or enlarged lymph nodes rather than complete anatomical nodal dissection. It was performed based on intraoperative surgeon judgment without adherence to an SLN mapping algorithm. All these patients were analyzed separately and were not considered part of the SLN cohort.

The small subgroup of patients who received neoadjuvant radiochemotherapy were included in the same predefined lymph node assessment categories according to the final intraoperative surgical strategy.

In cases of unilateral SLN mapping failure, side-specific lymph node assessment was performed on the non-mapped side according to the sentinel lymph node algorithm.

All retrieved lymph nodes were submitted separately for histopathological examination and ultrastaging, in accordance with institutional protocols. Mapping outcomes, including bilateral versus unilateral detection, anatomical distribution of SLNs and comparative performance of the two tracers, were recorded prospectively for analysis.

### 2.3. Data Collection

Clinical, intraoperative, and pathological data were extracted from the institutional electronic record system and organized into a structured database. Variables that were analyzed included:

- Patient demographics (age);
- Neoadjuvant treatment;
- Lymph node assessment—SLN (and technique) or SLNS;
- Number of lymph nodes retrieved;
- Number of positive lymph nodes retrieved;
- Location of SLN;
- Histological type;
- Grade of nuclear differentiation;
- Status of surgical margins;
- Lymphatic, vascular and perineural invasion;
- Radicality;
- Hormonal receptor positivity;
- Microsatellite instability (MSI);
- Cervical stroma invasion;
- Adjuvant treatment.

### 2.4. Study Endpoints

The primary endpoint of the study was lymph node assessment performance, evaluated through lymph node yield and nodal metastasis detection rate. Bilateral pelvic nodal assessment and the association between pathological T stage and lymph node involvement were analyzed as exploratory and descriptive outcomes.

### 2.5. Statistical Analysis

For the characterization of our cohort, both descriptive and inferential statistics were used. In each case, continuous variables were represented as average value ± standard deviation (SD) or median with interquartile range (IQR). On the other hand, categorical variables were expressed by absolute and relative frequencies.

Comparisons between groups were performed using the independent *t*-test for continuous variables, the Chi-square test or Fisher’s exact test for categorical variables, and the Kruskal–Wallis test for comparisons across the three lymph node assessment groups.

Statistical analyses were performed using IBM SPSS Statistics for Windows (Version 29.0) and Microsoft Excel, Microsoft 365 (Version 2407).

## 3. Results

### 3.1. Patient Demographics and Management Strategies

A total of 137 patients with endometrial carcinoma (with complete surgical and histopathological data) were included in the analysis. The median age was 62.5 years (mean ± SD: 62.4 ± 10.6).

In most cases, no neoadjuvant treatment (132/137, 96.4%) was administered. Neoadjuvant radio-chemotherapy was administered in five patients (3.6%) and was distributed across the study groups without a predominant allocation to any specific lymph node assessment technique. The majority of patients underwent simple hysterectomy (117/137, 85.4%), while radical hysterectomy was performed in only 20 cases (14.6%). Adjuvant therapy was administered to 85 patients (62.0%), while the other 52 patients (38.0%) did not receive any additional treatment. Among those who received adjuvant therapy, 45 patients were treated using brachytherapy alone, whereas 40 patients received a combination of external-beam radiotherapy, chemotherapy and brachytherapy.

### 3.2. Histopathological Characteristics

Endometrioid carcinoma was the predominant histologic type in the cohort, accounting for 136 cases (99.3%), while the remaining case consisted of clear cell carcinoma. Most of the tumors did not present any associated histologic variants (130/137, 94.9%); even if three cases had clear cell features, two had mucinous differentiation and two had serous differentiation. In terms of tumor grade, 56 tumors (40.9%) were G1, 55 (40.1%) were G2, and the other 26 (19.0%) were G3.

**Figure 2:**
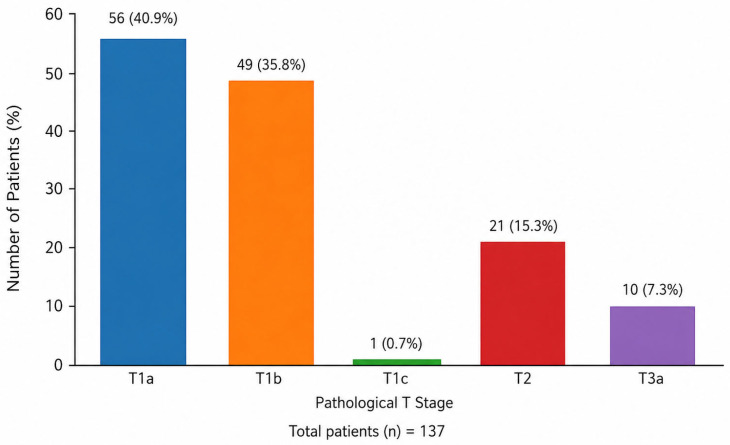
Distribution of patients according to pathological T stage.

The majority of patients presented with early-stage disease in accordance with pathological T staging (Figure 2). Pathological T1a was recorded in 56 patients (40.9%), T1b in 49 (35.8%), T2 in 21 (15.3%), and T3a in 10 (7.3%); one patient (0.7%) was classified as T1c.

**Figure 3:**
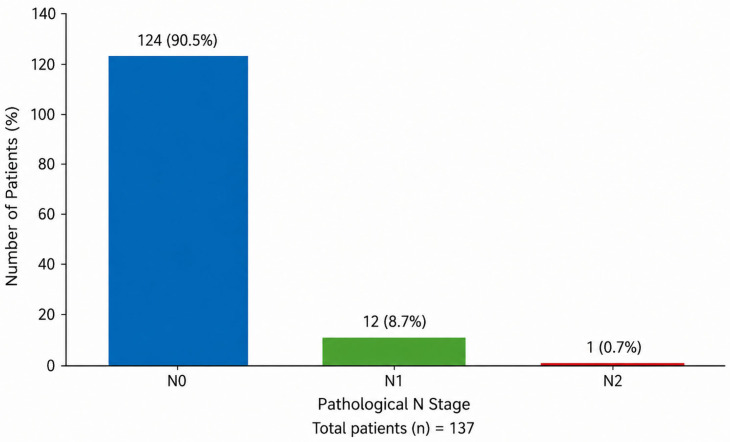
Distribution of patients according to pathological N stage.

Regarding lymph node status (Figure 3), the vast majority did not have any nodal involvement (N0, 124/137, 90.5%), with a small proportion exhibiting N1 (12/137, 8.7%) and N2 (1/137, 0.7%).

Surgical margins were negative in nearly every case, with R0 resection achieved in 136 patients (99.3%), while microscopic residual disease (R1) was documented in only one case (0.7%).

Cervical stromal invasion was present in 17/137 cases (12.4%). Lymphatic invasion was observed in 26 patients, vascular invasion in 3 cases, and perineural invasion in a single patient. Hormone receptor status was assessed in 131 patients, of whom 115 (87.8%) were positive and 16 (12.2%) were negative; data were missing in 6 patients. Microsatellite status was available for 104 of 137 patients. Among these, microsatellite stability (MSS) was observed in 79/104 (76.0%), while microsatellite instability-high (MSI-H) was identified in 25/104 (24.0%). Data were unavailable for 33 cases.

### 3.3. Lymph Node Assessment and Subgroup Statistical Analysis

Lymph node evaluation was performed either via SLNS in 51 patients or SLN mapping in 86 patients (Table 1). SLN mapping was carried out using BD in 45 cases and ICG in 41 cases. The most frequent SLN locations were bilateral ilio-obturator (44/137), bilateral external iliac (12/137), and bilateral internal iliac (12/137). Other combinations of unilateral or mixed iliac and obturator locations were less common. Most patients had no positive lymph nodes (123/137, 89.8%), while the remainder had between 1 and 5 positive nodes or micrometastases.

Globally, lymph node yield had a median of 6 nodes (interquartile range [IQR] 3–9), with an average of 7.5 ± 6.5 nodes (range 0–34). Lymph node yield differed noticeably between these three strategies (Table 1). Patients in the SLNS group had a median of 10 nodes removed (IQR 7–17.5; mean 12.5 ± 7.9), compared to 4 nodes (IQR 3–6; mean 4.4 ± 2.4) in the BD group and 4 nodes (IQR 2–7; mean 4.7 ± 3.1) in the ICG group. The Kruskal–Wallis test demonstrated that differences in lymph node yield across techniques were statistically significant (H = 50.8, *p* < 0.001).

Lymph node metastases (with at least one positive node) were identified in 13 of 137 patients, corresponding to an overall nodal involvement rate of 9.5%. In these cases, the number of involved nodes varied between 1 and 5, with a median value of 2 (IQR 1–3) and a mean of 2.2 ± 1.4.

The distribution of nodal involvement across pathological T categories was as follows: 0/56 node-positive (0%) for T1a, 5/49 node-positive (10.2%) for T1b, 0/1 node-positive (0%) for T1c, 4/21 node-positive (19.0%) for T2 and 4/10 node-positive (40.0%) for T3a. This showed a statistically significant increasing trend (chi-square test: χ^2^ = 19.1, *p* < 0.001) in nodal positivity with advancing T stage.

When nodal status was analyzed according to the lymph node assessment strategy, similar rates of nodal metastasis were observed across all three groups, as follows: 5/51 node-positive (9.8%) for SLNS, 4/45 node-positive (8.9%) for BD, and 4/41 node-positive (9.8%) for ICG. There was no statistically significant difference regarding nodal positivity rates between these techniques (chi-square test: χ^2^ = 0.03, *p* = 0.99), with very low event rates resulting in limited statistical power and wide uncertainty around comparative estimates.

According to the recorded sentinel node localization, bilateral detection of pelvic nodes was achieved in 101 of 137 patients (73.7%), while the other 36 (26.3%) had unilateral or incomplete pelvic mapping. When stratifying by technique, 47/51 patients (92.2%) had bilateral detection in the SLNS group, 33/45 (73.3%) in the BD group, and 21/41 (51.2%) in the ICG group, as represented in Table 1. When comparing the two sentinel-focused techniques, BD mapping showed a significantly higher likelihood of bilateral detection than ICG (33/45 vs. 21/41): OR = 2.62 (95% CI: 1.06–6.45), *p* = 0.045 (Fisher’s exact test).

## 4. Discussion

### 4.1. Interpretation of the Main Findings

In our cohort of 137 patients with histologically confirmed endometrial carcinoma, the population was predominantly diagnosed at an early stage. The median age of 62.5 years is consistent with the epidemiological profile of endometrial cancer, reinforcing the representativeness of the studied cohort.

From a therapeutic point of view, the reduced rate of neoadjuvant treatment is in accordance with standard clinical practice in endometrial carcinoma, where surgery of primary intention remains the cornerstone of treatment. The predominance of simple hysterectomy further reinforces the early-stage distribution noticed in the cohort, while the selective use of radical hysterectomy and adjuvant therapies appears linked to pathological risk factors.

Histopathologically, the predominance of endometrioid carcinoma and low-to-intermediate tumor grades illustrates the generally favorable behavior of the tumors included in this study. The majority of cases showed extension limited to the uterus (pT1–pT2), with decreased rates of nodal involvement and achievement of mainly negative surgical margins, supporting the overall good oncological control achieved via surgery. The relatively low frequency of cervical stromal invasion and lymphovascular invasion further confirms the predominance of low-risk neoplasms within the cohort. Moreover, the increased proportion of hormone receptor-positive tumors and the distribution of microsatellite instability status are aligned to the molecular and prognostic patterns of endometrial cancer.

A key finding of the current study is related to lymph node assessment strategies. Even if lymph node counts are determined postoperatively by histopathological examination and might be influenced by tissue composition and processing, the extent of surgical resection inherently differs between SLNS and SLN mapping techniques. Thus, differences in nodal yield across these strategies should be interpreted in the context of procedural intent rather than as a direct measure of technique performance. Furthermore, this fact did not imply a higher detection rate of nodal metastases. Noticeably, nodal positivity rates were almost similar across SLNS, BD-guided SLN mapping, and ICG-guided mapping, showing similar observed nodal staging outcomes across techniques within the limits of this observational study. These results further support the growing body of evidence that SLN mapping represents a reliable alternative to systematic SLNS in appropriately selected patients. Given the small number of nodal metastasis events, comparisons of nodal positivity rates between groups should be interpreted descriptively rather than as evidence of diagnostic equivalence.

Differences between the multiple techniques were observed with respect to bilateral pelvic mapping. BD mapping achieved a significantly higher bilateral detection rate compared to ICG, underscoring a potential variability related to tracer choice, institutional protocols and surgical experience, but should be interpreted cautiously. This finding is probably influenced by the observational nature of the study, potential differences regarding case allocation over time, variability in surgical workflow, and the inherent learning curve associated with the adoption of fluorescence-guided SLN mapping. Hence, this result should not be regarded as evidence of superiority of one tracer over the other, but rather as a reflection of real-world institutional practice.

What is more, despite differences regarding mapping performance and nodal yield, the comparable rates of nodal metastasis across different techniques illustrate that reduced nodal dissection does not appear to influence staging accuracy in this cohort.

Additionally, the strong and statistically significant association between advancing pathological T stage and increasing nodal involvement validate tumor depth and local extension as critical determinants of metastatic risk. This finding strengthens the importance of accurate assessment of the primary tumor for tailoring both surgical staging and adjuvant treatment strategies.

Overall, the results suggest that in a predominantly early-stage population of endometrial cancer with endometrioid histology, SLN mapping, despite yielding fewer nodes, provides nodal staging information that is comparable to SLNS, while pathological tumor stage remains the main predictor of nodal dissemination.

### 4.2. Alignment with the Existing Literature

A large population-based study (SEER) by Nahshon et al. [16] included 41,411 patients who were diagnosed with endometrial cancer between 2010 and 2019, and compared SLN sampling with full LND. After propensity score matching for a few parameters including age, histology, grade, stage, and adjuvant treatment, 6019 patients were distributed to each group, with a median follow-up period of 16 months. Overall survival was not inferior in the SLN group; in fact, 1-year survival turned out to be significantly better compared with full LND (HR 1.61, 95% CI 1.17–2.21). Subgroup analysis indicated a clear benefit in terms of survival for SLN sampling in low-grade endometrioid tumors (grade 1 and 2), whereas survival was equivalent between the two techniques in high-grade tumors. Notably, nodal metastases were discovered less frequently in low-grade tumors (7%) compared to high-grade tumors (17%), indicating that a selective and less morbid approach such as SLN does not compromise oncologic safety.

A single-institution retrospective study by Holtzman et al. [17] assessed outcomes in 189 cases with diagnosis of high-risk endometrial cancer, via comparing SLN mapping with pelvic ± para-aortic LND. Overall, 46 patients underwent bilateral SLN mapping, while the other 143 underwent extensive LND, with no statistically significant differences in terms of baseline clinicopathologic parameters between groups. Three-year progression-free survival was almost identical (71.1% SLN vs. 71.3% LND), and SLN evaluation was not associated with increased risk of recurrence, even after multivariable adjustment. Even though overall survival was lower in the group with SLN, this difference disappeared after adjustment of age, surgical approach and adjuvant treatment. Globally, this reinforces that SLN mapping is an oncologically safe alternative to full LND in cases with high-risk endometrial cancer, without influencing progression-free or adjusted overall survival.

A retrospective multicenter study conducted by Capozzi et al. [18] included 237 patients with diagnosis of high-risk endometrial cancer (ESGO/ESTRO/ESP 2021 classification) to compare SLN biopsy alone with systematic pelvic LND. The median follow-up period was 31 months, and corresponding rates of recurrence and mortality equal to 16% and 8% were low. Disease-free survival and overall survival were similar between the SLN and LND groups (DFS: 85.2% vs. 82.8% and OS: 91.3% vs. 92.6%), with no statistically significant differences. Importantly, even in patients who presented nodal metastases, survival outcomes remained comparable between the two methods. These findings support that SLN mapping alone is oncologically safe in patients with high-risk endometrial cancer, and that systematic LND does not bring survival benefits.

In a single-center cross-sectional observational study conducted by García-Pineda et al. [19], SLNB was compared to LND in terms of quality of life in patients with early-stage endometrial cancer. Impairment of the physical functioning turned out to be lower in the SLNB group (8.2% vs. 25%, *p* = 0.031). Additionally, patients who underwent SLNB complained of fewer symptoms, such as sleep disturbance (4.9% vs. 27.6%, *p* < 0.01), pain (1.6% vs. 13.8%, *p* = 0.019), and dyspnea (0% vs. 10.3%, *p* = 0.011). In addition, sexual quality of life was also assessed, and the scores were consistently better in the SLNB cohort. Overall, SLNB was associated with improved postoperative well-being.

Using the National Cancer Database, Albright et al. [20] realized a large retrospective cohort study with the aim of assessing perioperative outcomes and disparities in the use of SLNB in minimally invasive staging of early-stage endometrial cancer. A large cohort of 67,365 patients was analyzed, and an increase in SLNB utilization was observed (from 2.8% in 2012 to 16.3% in 2016). Notably, SLNB was linked to a lower risk of conversion to open surgery when compared with LND (1.03–1.40% vs. 2.80%), not only in intention-to-treat (OR 0.53, 95% CI 0.39–0.72), but also in per-protocol analyses (OR 0.49, 95% CI 0.32–0.75). What is more, SLNB was associated with a lower risk of hospital stay exceeding one day (OR 0.51) and a lower rate of unplanned readmissions (OR 0.52). Hence, it turns out to be a less invasive approach, with enhanced perioperative outcomes.

### 4.3. Strengths of the Current Study

The current study has several notable strengths. Its prospective observational design and the inclusion of consecutive patients decrease selection bias and strengthen the reliability of the findings. The study was conducted in a high-volume tertiary center with expertise in gynecologic oncology, allowing for standardized surgical management and pathological assessment, which increases the internal validity of the results.

Second, the comparisons across multiple lymph node assessment strategies (SLNS and SLN mapping using two different tracers) provide a comprehensive evaluation of nodal staging techniques in daily clinical practice.

Third, the availability of complete histopathological and surgical data for the vast majority of items (which were prioritized) enabled an extensive characterization of tumoral features and prognostic factors, including pathological staging and status of lymph nodes.

Finally, the extended period of inclusion and relatively large number of patients included in the cohort (in the case of a single-center prospective study) support the representativeness of the population and the applicability of the results into routine clinical settings.

Collectively, all these strengths contribute to an organized methodological framework and bring valuable evidence that supports the results concerning the role of SLN mapping in the surgical staging of endometrial carcinoma.

### 4.4. Limitations of the Current Study

A few limitations of the current study should be acknowledged. First, even though the study was designed in a prospective and observational manner, it was conducted at a single tertiary referral center, which might limit the generalizability of the findings across other institutions, with different patient populations, surgical expertise or even lymph node mapping protocols. Institutional experience and surgeon proficiency, particularly with SLN detection techniques, can influence detection rates and nodal yield.

Second, despite the relatively long inclusion period of the patients, the overall sample size remains moderate and the number of cases with lymph node metastases was small. This limited event rate might decrease the statistical power to detect differences in nodal positivity between lymph node evaluation strategies and further restrict the ability to perform more subtle subgroup analyses.

Third, heterogeneity across lymph node assessment techniques represents another limitation. SLN mapping was performed using different tracers (BD and ICG) and SLNS was not standardized in terms of anatomical extent or nodal count. These variations could have contributed to the differences regarding nodal yield and bilateral detection rates, potentially introducing procedural bias.

Fourth, even if the study focused on surgical and histopathological outcomes, long-term oncologic endpoints such as disease-free survival, overall survival and patterns of relapse were not assessed. Consequently, the oncologic equivalence of the lymph node assessment techniques is not definitive.

Fifth, the non-randomized allocation of patients to different nodal assessment strategies (partially influenced by surgeon judgment and institutional practice) may have introduced selection bias and limited baseline comparability between groups.

Sixth, the relatively low number of node-positive cases limited the statistical power to detect differences in nodal metastasis rates between groups. Thus, the absence of statistically significant differences should not be regarded as evidence of equivalence or comparable diagnostic accuracy between lymph node assessment strategies.

Seventh, because of the limited number of outcome events and sample size, multivariable adjustment for potential confounders (including tumor grade, pathological stage, histology, lymphovascular invasion, surgical approach, and treatment-related factors) was not feasible, which might introduce residual confounding.

Eight, an additional limitation relates to the heterogeneity of histopathological processing, which reflects real-world institutional practice. Even though all lymph node specimens were assessed according to standardized local protocols, minor variations with respect to ultrastaging procedures (including sectioning depth, number of serial levels, and selective use of immunohistochemistry) may have been present over the long study period. These differences are inherent to routine clinical workflows in a high-volume tertiary center and are unlikely to have introduced systematic bias across groups. However, they may contribute to subtle variability in the detection of low-volume metastases. But such variability does not influence the primary comparisons of nodal yield and macroscopic nodal positivity, which remain robust across the study cohorts.

Finally, a small number of patients were excluded due to incomplete medical records or absence of lymph node assessment, which were addressed in the patient selection process.

Altogether, these limitations should be considered when interpreting the results, and they emphasize the need for larger, multicenter studies with standardized surgical protocols and long-term follow-up strategies to further validate the role of different lymph node assessment techniques in endometrial cancer.

## 5. Conclusions

In this prospective observational study, most patients with endometrial carcinoma presented with early-stage and predominantly endometrioid disease and were further managed according to the current standard surgical and adjuvant treatment protocols. Lymph node involvement was relatively rare and strongly associated with advancement of pathological T stage, validating tumor extent as a key determinant of metastatic risk.

Even though SLNS resulted in a significantly higher lymph node yield compared to SLN mapping, this did not imply higher rates of nodal metastasis detection. SLN mapping, using either BD or ICG demonstrated comparable observed nodal positivity rates to SLNS (within the limitations of this observational study), supporting its role as a reliable instrument for lymph node evaluation. Differences in terms of bilateral detection rates were observed between the two tracers. However, these variations did not influence overall nodal positivity rates.

Altogether, SLN mapping showed similar nodal positivity rates in this cohort; however, larger multicenter, standardized prospective studies are required to confirm comparable performance with other lymph node assessment strategies.

## Figures and Tables

**Table 1:** Surgical and nodal assessment outcomes by technique.

Variable	SLN–BD (*n* = 45)	SLN–ICG (*n* = 41)	SLNS (*n* = 51)
Lymph node yield (median)	4	4	10
Lymph node yield (mean ± SD)	4.4 ± 2.4	4.7 ± 3.1	12.5 ± 7.9
Total nodal metastasis rate	4/45 (8.9%)	4/41 (9.8%)	5/51 (9.8%)
Overall nodal positivity (group %)	8.9%	9.8%	9.8%
Bilateral detection rate	33/45 (73.3%)	21/41 (51.2%)	47/51 (92.2%)
Unilateral/incomplete mapping	12/45 (26.7%)	20/41 (48.8%)	4/51 (7.8%)

SLN—sentinel lymph node; BD—blue dye; ICG—indocyanine green; SLNS—selective lymph node sampling.

## Data Availability

Data are available upon reasonable request from the corresponding authors.
